# A *De Novo*-Assembly Based Data Analysis Pipeline for Plant Obligate Parasite Metatranscriptomic Studies

**DOI:** 10.3389/fpls.2016.00925

**Published:** 2016-07-11

**Authors:** Li Guo, Kelly S. Allen, Greg Deiulio, Yong Zhang, Angela M. Madeiras, Robert L. Wick, Li-Jun Ma

**Affiliations:** ^1^Department of Biochemistry and Molecular Biology, University of Massachusetts Amherst, Amherst, MAUSA; ^2^Stockbridge School of Agriculture, University of Massachusetts Amherst, Amherst, MAUSA

**Keywords:** metatranscriptomics, RNA-seq, bioinformatics pipeline, *de novo* assembly, host–pathogen interaction, obligate biotroph, downy mildew

## Abstract

Current and emerging plant diseases caused by obligate parasitic microbes such as rusts, downy mildews, and powdery mildews threaten worldwide crop production and food safety. These obligate parasites are typically unculturable in the laboratory, posing technical challenges to characterize them at the genetic and genomic level. Here we have developed a data analysis pipeline integrating several bioinformatic software programs. This pipeline facilitates rapid gene discovery and expression analysis of a plant host and its obligate parasite simultaneously by next generation sequencing of mixed host and pathogen RNA (i.e., metatranscriptomics). We applied this pipeline to metatranscriptomic sequencing data of sweet basil (*Ocimum basilicum*) and its obligate downy mildew parasite *Peronospora belbahrii*, both lacking a sequenced genome. Even with a single data point, we were able to identify both candidate host defense genes and pathogen virulence genes that are highly expressed during infection. This demonstrates the power of this pipeline for identifying genes important in host–pathogen interactions without prior genomic information for either the plant host or the obligate biotrophic pathogen. The simplicity of this pipeline makes it accessible to researchers with limited computational skills and applicable to metatranscriptomic data analysis in a wide range of plant-obligate-parasite systems.

## Introduction

Many devastating agricultural plant diseases are caused by obligate parasitic microbes. These parasites include fungi, such as rusts (Hulbert and Pumphrey, 2014) and powdery mildews (Glawe, 2008), and oomycetes such as downy mildews (Yarwood, 1956; Perfect and Green, 2001). Obligate parasites are typically recalcitrant to axenic culture, resistant to genetic manipulation, and require living host plants to survive and propagate (Glazebrook, 2005; Bindschedler et al., 2016). These characteristics make it challenge to study the pathogenesis using conventional genetics and molecular biology, thus impeding the development of effective control strategies.

RNA sequencing (or RNA-seq) is a powerful next-generation sequencing technology that allows researchers to characterize and quantify the active transcriptome of organisms from which RNA can be extracted (Ozsolak and Milos, 2011). Numerous transcriptomic studies have applied RNA-seq to plants, plant pathogens, or mixed host–pathogen samples (metatranscriptomics). Metatranscriptomics has been used to explore the interaction between *Phytophthora infestans* (the late blight causal organism) and a susceptible tomato cultivar (Solanum lycopersicum, cv. M82), as well as Septoria tritici blotch (STB) of wheat caused by Zymoseptoria tritici (Grandaubert et al., 2015; Zuluaga et al., 2016). However, in each of these pathosystems data from one or both of the organisms could be compared to a reference genome.

Here, we developed a comprehensive computational pipeline integrating NGS data processing, *de novo* assembly, host and pathogen transcript separation, functional annotation, and differential gene expression analysis without the need for a reference genome (see the detailed protocol in Supplementary Material accompanying this article). The pipeline is compatible with a broad range of plant-pathogen systems. In this study, we have tested the pipeline using metatranscriptomic data of sweet basil (*Ocimum basilicum*) and its obligate downy mildew parasite *Peronospora belbahrii*, both lacking a sequenced genome.

Downy mildew of sweet basil (*O. basilicum*) is caused by *P. belbahrii*, an obligate biotrophic oomycete pathogen that infects the plant mesophyll tissue under cool, humid conditions (Garibaldi et al., 2007). Characteristic symptoms of infected leaves include interveinal chlorosis with gray, downy sporulation on the abaxial surface of leaves (Belbahri et al., 2005; Garibaldi et al., 2007; Koroch et al., 2013). In the US regions affected by the disease, growers have reported up to 100% crop loss with estimated financial losses in the tens of millions of dollars (Roberts et al., 2009; Wyenandt et al., 2015). Chemical controls for basil downy mildew have variable efficacy, and are vulnerable to the development of pathogen resistance (Pyne et al., 2014). Both Sweet basil and *P. belbahrii* have only limited available genomic resources, despite the use of sweet basil in volatile oil production research (Gang et al., 2001) and the recent sequencing of nine oomycete plant pathogen genomes (Pais et al., 2013).

Using our computational pipeline, we have identified nearly 3,000 candidate *P. belbahrii* genes that are expressed in planta. We also identified over 1,000 *O. basilicum* genes expressed more than 4 times higher during infection as compared to the control. Most interestingly, these genes are enriched for biological processes such as biotic and abiotic stress responses, demonstrating the power of RNA-seq even under the condition that biological replicates are not available. Using this set of data, we have demonstrated the utility of our metatranscriptomic analysis pipeline for studying plant and obligate parasite interactions.

## Results

### Metatranscriptome Sequencing and Assembly

**FIGURE 1:**
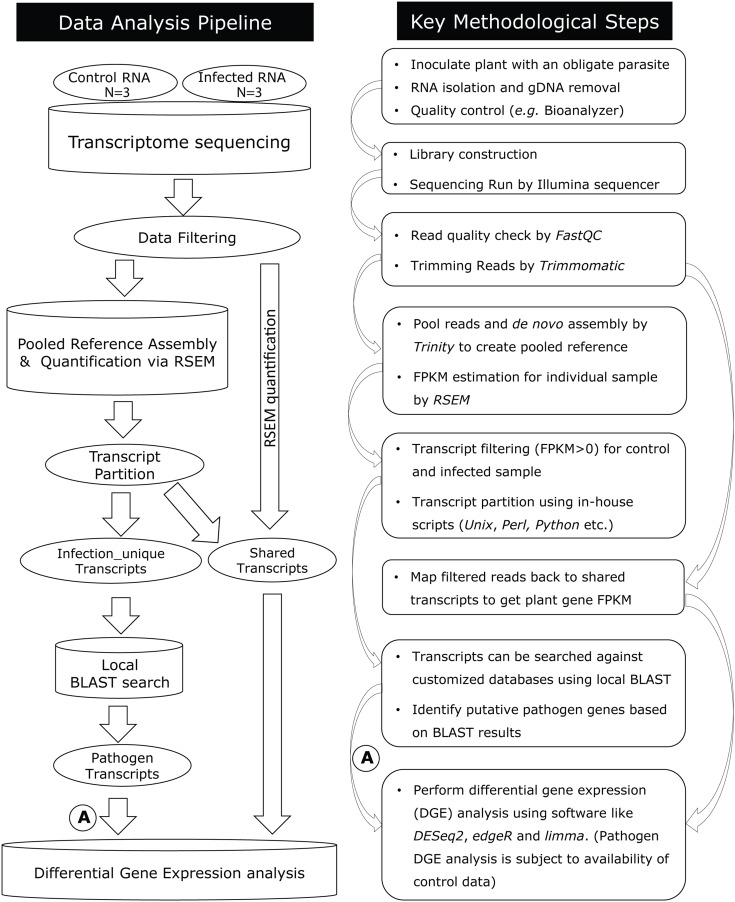
**Diagram summarizing the data analysis pipeline to analyze host–pathogen metatranscriptomes and key methodological steps.** A step-by-step protocol of this pipeline is available (**Supplementary Material**). After quality filtering, RNA-seq reads are assembled *de novo* using Trinity. For pathogen transcript discovery, a “pooled reference” is assembled combining control and infected plant reads, which are further divided into control-unique, infected-unique, and shared groups. For plant differential gene expression analysis, shared transcripts are used as a reference, against which control and infected reads are mapped by RSEM. A = DGE analysis for pathogen transcripts are subject to availability of a reference sample.

This computational analysis pipeline was designed to enable metatranscriptomic data analysis, downstream transcript discovery, and expression analysis in plant-obligate-parasite pathosystems (**Figure 1**; **Supplementary Material**). The pipeline includes quality control, *de novo* assembly, transcript quantification, transcript partition, BLAST search, annotation, and differential gene expression analysis.

**FIGURE 2:**
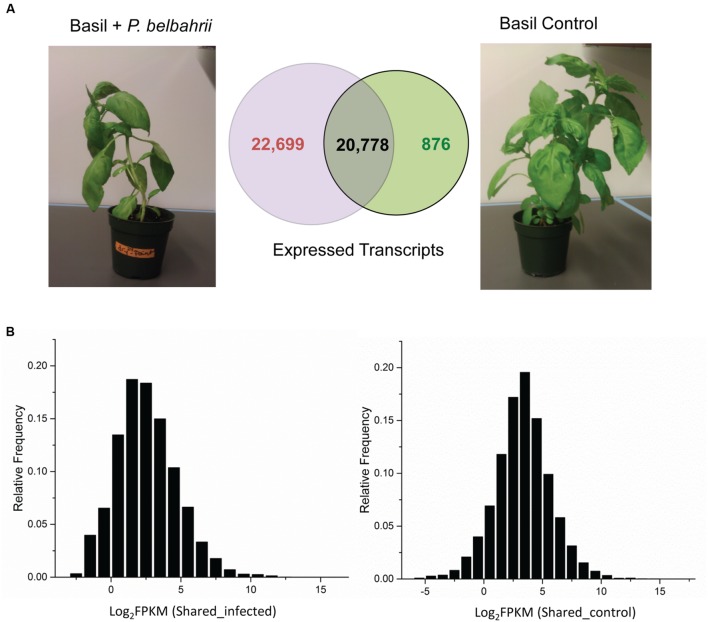
**Transcript comparison between control and infected plants. (A)** Total number and overlap between transcripts discovered in infected sweet basil 5 dpi (Basil + *Peronospora belbahrii*) and uninoculated plants (Basil Control). **(B)** Distribution of the base 2 logarithm of FPKM (fragment per kilobase of exon per million fragments mapped) for shared transcripts under infected (left) and control (right) conditions. Note that shared transcripts (plant genes) have a higher (approximately twice as much) average read coverage in the control condition compared to the infected condition.

To demonstrate the application of this pipeline, we generated a test set of RNA-seq data from sweet basil infected with *P. belbahrii* (**Figure 2**). Total RNA was purified from one uninoculated basil plant (control) and one infected with *P. belbahrii* 5 days post-inoculation (dpi). The purified RNA product was sequenced using the Illumina Hiseq (see MATERIALS AND METHODS). In total, the RNA-seq experiment generated 24 million (M) and 37M paired-end reads from the control and infected plant, respectively. After removing low quality reads and trimming poor quality bases, a total of 22M and 35M paired-end reads were retained for the control and infected samples, respectively. High quality filtered reads were then pooled and assembled *de novo* using Trinity (Grabherr et al., 2011), yielding a total of 44,643 genes, which were designated the “pooled reference.”

To calculate transcript abundance, filtered infected and control reads were mapped back to the pooled reference separately. FPKM (fragment per kilobase of exon per million fragments mapped), a numerical value representing relative gene expression, was estimated using RSEM (RNA-seq by expectation maximization; Li and Dewey, 2011). Comparison of transcripts from infected and control plant samples placed all transcripts into one of three categories: control-unique, shared, and infected-unique transcripts (see next section for details). Based on the FPKM distribution of shared transcripts, the average coverage of RNA-seq reads was approximately 12X for the uninoculated plant and 6X for the infected plant (**Figure 2**), despite the fact that more sequence reads were generated for the infected sample. The two-fold difference could be attributed to the different composition of sequence reads in the infected sample (mixture of host and pathogen reads) and the control sample (solely host reads). Indeed, the infected sample had almost twice the number of unique transcripts compared to the control sample (discussed below).

### *P. belbahrii* Transcript Discovery

To differentiate basil and *P. belbahrii* genes, we collected 43,477 and 21,654 genes with non-zero expression values from infected and uninoculated basil, and further divided them into three categories: genes unique to uninoculated basil (876), genes unique to infected basil (22,699), and genes shared by infected and uninoculated basil (20,778). Genes uniquely present in the infected sample are likely composed of *P. belbahrii* genes and basil genes only expressed during infection. This division narrowed the search for candidate *P. belbahrii* genes to within a smaller subset of 22,699 genes.

**FIGURE 3:**
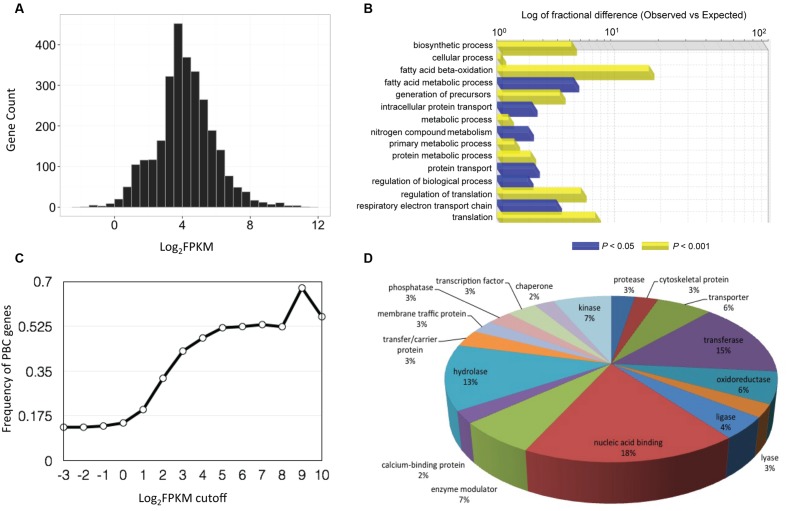
**Basil downy mildew (*P. belbahrii*) gene discovery and functional annotations. (A)** Gene count distribution of the base 2 logarithm of FPKM (Log2FPKM) among 2,934 *P. belbahrii* candidate (PBC) genes. **(B)** Frequency of PBC genes (0–1) in infected-unique transcripts filtered by series of Log_2_FPKM threshold (-3 to 10). **(C)** Functional classification of highly expressed PBC genes (Log_2_FPKM > 7), performed by PANTHER (Protein Analysis Through Evolutionary Relationships) gene list analysis using *Phytophthora infestans* homologs. Blue and yellow bars represent significantly enriched biological processes under FDR (false discovery rate) cutoff of 0.05 and 0.001, respectively. **(D)** A pie-chart of protein class analysis for all PBC genes that have homologs in *P. infestans*.

To identify putative *P. belbahrii* genes, we performed a local BLAST search of the 22,699 infection-unique genes against a customized oomycete genome database (see MATERIALS AND METHODS). Using a stringent *E*-value threshold (*E*-value < 1e - 50), we identified 2,934 (13%) oomycete homologous genes, defined as PBC (*P. belbahrii* candidate) genes. PBC genes had wide ranging FPKM values, ranging from less than 1 to greater than several thousand (**Figure 3A**). Interestingly, increasing the FPKM cutoff to 512 FPKM was used, 60% (27) were PBC genes (**Figure 3B**).

PBC genes have a wide range of biological functions. Among PBC genes 2,711 (92%) have a homolog (sequence similarity > 60%, *E*-value < 1e - 20) in the genome of *P. infestans*, a well-studied plant pathogenic oomycete. PANTHER (Protein Analysis Through Evolutionary Relationships; Thomas et al., 2003) analysis of *P. infestans* homologs suggests that many homologs code for nucleotide-binding proteins, transferases, hydrolases, enzyme modulators, oxidoreductases, proteases, lyases, kinases, and transcription factors (**Figure 3D**). Interestingly, all four histone core proteins and components of ribosomal complexes are among the most highly expressed PBC genes. Gene Ontology (GO) enrichment analysis showed that highly expressed PBC genes (Log_2_FPKM > 7) were enriched for fatty-acid oxidation, translation, regulation of translation, and other biosynthetic processes (**Figure 3C**), indicating that *P. belbahrii* is physiologically active.

We have also identified several PBC transcripts that are homologous to known virulence factors in *P. infestans*, including the secreted RXLR effectors (Kamoun, 2006). Specifically, we identified two PBC genes encoding putative *P. belbahrii* RXLR effectors, named PbRX1 (Trinity assembly: comp66055_c2) and PbRX2 (Trinity assembly: comp59755_c0), homologous to PITG_03155 (*E*-value: 9e - 101) and PITG_09585 (*E*-value: 2e - 124) in *P. infestans*, respectively. Whether the two *P. belbahrii* RXLR effectors contribute to downy mildew pathogenesis as typical RXLR proteins remains to be confirmed. A comprehensive expression study can be implemented to monitor the expression profiles of these candidate effectors and to identify functional importance during host–pathogen interaction. Both housekeeping proteins and these candidate RXLR effectors could be used to develop biomarkers to study pathogen population structure and to monitor the presence of pathogen in field or greenhouse production.

### Basil Genes Responding to *P. belbahrii* Infection

Understanding that some plant genes are only turned on in responding to *P. belbahrii* infection, we searched the infection-unique transcripts against the Plant genome database PlantGDB^1^. A search with high stringency (*E*-value > 1e - 50) identified 1,667 or 40 infection-unique transcripts only mapped to plant genes with a FPKM value greater than 0 or greater than 10, respectively. Among the 40 plant transcripts with high FPKM values, 30 of them have homologous sequences in *Arabidopsis* genome and the most significantly enriched GO annotation is “response to external stimulus” (*P* = 1.5e - 05 with a false discovery rate of 0.00089).

Important, but still relatively smaller proportion of infection-unique transcripts (<2% transcripts with a FPKM value greater than 10) are plant genes, which indicates that most plant genes are expressed in both control and infected samples. Genes expressed differently during infection can also be important to understand plant defense against parasites. Various software packages are available for differential gene expression analysis such as edgeR, DESeq, and limma. In our pipeline, we have implemented edgeR for the discovery of differentially expressed genes using data with biological replicates (Supplementary Protocol).

**FIGURE 4:**
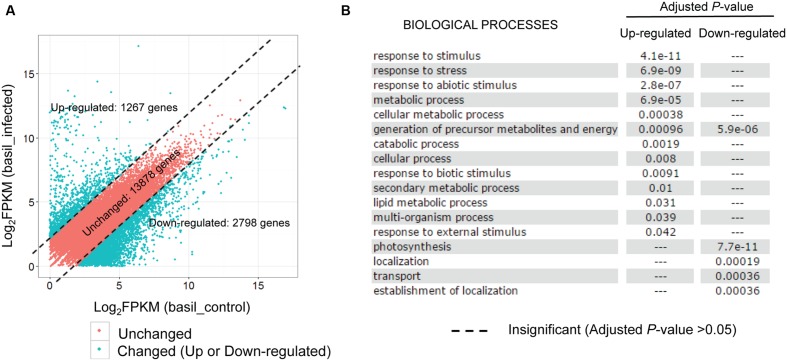
**Sweet basil genes are regulated in response to downy mildew infection. (A)** Scatterplot of basil gene expression levels (Log_2_FPKM) detected in both control and infected conditions. Note that plotted are only genes with FPKM larger than 1 for both conditions. Red points, representing 13,878 genes with comparable expression levels in two conditions (unchanged), are separated by dashed lines from blue dots representing 1,267 and 2,798 transcripts that are at least four times higher or lower in the infected plants compared to the control sample. **(B)** GO enrichment analysis using *Arabidopsis thaliana* homologs of the 1,267 up-regulated and 2,798 down-regulated genes using AgriGO. Each row shows whether a biological process is either significantly or insignificantly enriched in up-regulated and down-regulated genes, as indicated by an adjusted *P*-value (cutoff: 0.05).

Exploring the test datasets generated from the sweet basil and its obligate biotrophic pathogen downy mildew *P. belbahrii* pathosystem, we created a “shared reference” transcript set using the 20,778 genes present in both the control and the infected plants. The expression of each basil gene was then re-estimated using RSEM by mapping control and infected plant reads to the shared reference independently. After applying an FPKM threshold (FPKM > = 1), 17,943 transcripts were used for differential gene expression analysis. Lacking biological replicates, we wanted to be stringent in selecting plant genes potentially differentially expressed under pathogen challenge. Using a fourfold change cutoff, we identified 1,267 (7.0%) up-regulated, and 2,798 (16.6%) down-regulated transcripts in inoculated versus uninoculated plants, respectively (**Figure 4A**). Local BLAST (sequence similarity >60% and *E*-value < 1e - 20) against the *Arabidopsis thaliana* genome identified 565 up- and 523 down-regulated *A. thaliana* homologs. Interestingly, GO enrichment (adjusted *P*-value < 0.05) of these *A. thaliana* homologs using AgriGO (Du et al., 2010) suggested distinct biological functions for up- versus down-regulated transcripts. While up-regulated transcripts were significantly enriched for biotic and abiotic stress response, response to external stimuli, and metabolic processes, down-regulated transcripts were significantly enriched for photosynthesis, generation of precursor metabolites, energy production, transport, and localization (**Figure 4B**). Distinct GO term enrichment reflects a metabolic physiological switch from an active growth to an energy preservation response under biotic stress conditions.

Among the up-regulated sweet basil genes, we found several with high fold changes ranging from 15 to 40. These highly up-regulated genes included one beta-glucanase gene (BG3), two lipoxygenases genes (LOX1 and LOX2), the WRKY transcription factor WRKY33, the heat-shock protein HSP70-1, a cytochrome P450 (CYP81D1), and the elicitor-activated gene ELI3-1 (**Table 1**). Many of these genes have well-characterized roles in the plant defense response against various pathogens, including BG3, which has been reported to respond to infection by the bacterial pathogen *Pseudomonas syringae* pv. maculicola (Dong et al., 1991). LOX1 and LOX2 are involved in the jasmonic acid response signaling pathway triggered by pathogen infection (Melan et al., 1993; Bell et al., 1995). WRKY33 has been reported as a key transcription factor induced by fungal (Zheng et al., 2006) and oomycete infections (Merz et al., 2015). In addition to homologs of well-characterized genes, 6 receptor-like kinases, a mitogen-activated protein kinase (AtMPK4), and 17 transcription factors likely involved in pathogen sensing and downstream signaling were identified, suggesting that fundamental plant defense-signaling pathways are induced during downy mildew infection. These defense genes could be useful during routine plant screening for disease prior to visible symptom development.

**Table 1:** Sweet basil biotic stress response genes induced by *Peronospora belbahrii* infection and their putative functional annotation.

Assembled Basil genes	*Arabidopsis* genes	TAIR Gene_ID	BLAST* *E*-value	Log_2_FC	Functional annotation
comp48041_c2	BG3	AT3G57240.1	5.00e - 29	5.45	β-1,3-glucanase 3
comp51245_c2	HSC70-1	AT5G02500.1	2.00e - 77	2.42	Heat shock cognate protein 70-1
comp49399_c0	WRKY33	AT2G38470.1	4.00e - 25	5.55	WRKY transcription factor
comp50532_c0	CYP81D1	AT3G28740.1	1.00e - 73	3.29	Cytochrome p450
comp50896_c0	AGB1	AT4G34460.4	4.00e - 53	3.03	Heterotrimeric G-protein beta subunit
comp47595_c0	ELI3-1	AT4G37980.1	3.00e - 32	4.12	Elicitor-activated gene 3-1
comp40515_c0	NHL25	AT5G36970.1	4.00e - 65	2.20	NDR1/HIN1-like protein
comp51070_c3	HSPRO2	AT2G40000.1	6.00e - 62	2.44	*Arabidopsis* ortholog of sugar beet HS1 pro-1 2
comp46635_c0	LOX1	AT1G55020.1	3.00e - 40	2.46	Lipoxygenase 1
comp35556_c0	LOX2	AT3G45140.1	2.00e - 76	2.02	Lipoxygenase 2
comp50754_c0	ATMRP4	AT2G47800.1	9.00e - 62	2.42	*A. thaliana* multidrug resistance-associated protein 4
comp50993_c1	ATOSM34	AT4G11650.1	4.00e - 66	2.79	Osmotin-like protein osmotin 34
comp48666_c0	NHO1	AT1G80460.1	3.00e - 50	2.42	Non-host resistance to *P. s. phaseolicola* 1

TAIR, The *Arabidopsis* Information Resource; Log_2_FC, the base 2 logarithm of fold change.

* The *E*-value cut-off used for the BLAST search is 1e - 20.

## Discussion

We have developed a computational pipeline composed of freely available software for analyzing metatranscriptomic data. This pipeline has clear advantages for analyzing systems without reference genomes, and is friendly designed to support researchers lacking bioinformatic training. Using this pipeline, we identified about 3,000 actively transcribe genes from *P. belbahrii*, when this obligate downy mildew pathogen infecting its host sweet basil at 5 dpi. This is consistent with reference genome based RNA sequencing of Hyaloperonospora arabidopsidis, which was shown to express 2,293 and 6,858 genes in planta at 1 and 3 dpi (Asai et al., 2014). These transcripts covered a wide range of GO functions including nucleic acid binding, transferases, hydrolases, calcium binding, transcription factors, and chaperones. We also identified two homologs to *P. infestans* RXLR effector proteins.

In addition to the identification of pathogen transcripts, we tentatively discovered 4,065 differentially expressed candidate plant transcripts. The identification of up-regulated transcripts involved in biotic and abiotic stresses and the response to external stimuli likely indicates a host response to pathogen attack. Fundamental to the success of this pipeline is the inclusion of a sample completely lacking pathogen nucleic acid (uninoculated control). This control reference allows for the identification of both host transcripts in response to pathogen attack and transcripts unique to infected plants, of which pathogen transcripts are a subset. Transcripts assembled from either the control or the infected samples may include sequences from commensal microbes present in soil samples. As these transcripts should have similar presentation in both samples, comparative study between two data sets could remove most sequences belonging to these categories.

To make this pipeline user-friendly, we have simplified the steps involved in the data analysis. The use of pooled reads from both samples for the generation of the initial reference assembly adds one additional step, but removes a complicated downstream BLAST step normally needed when data sets are mapped to separate references. This process makes the identification of shared, control-specific, and infection-specific transcripts significantly easier. The subsequent use of the shared transcript reference to map both the control sample and the infected sample allows for more accurate FPKM normalization, fixing an error generated when using the pooled reference and leading to a more precise calculation of host plant differential gene expression.

To achieve greater levels of statistical confidence, it is advised that a minimum of three biological replicates per condition be used. Biological replicates strengthen differential gene expression analysis between samples. Additionally, multiple replicates aid in the discovery of pathogen and host genes with low FPKM values, which are potentially overlooked when using a single data set. A protocol for the use of this pipeline with multiple replicates is available in the **Supplementary Material**.

This pipeline has been effective in analyzing the interaction between two organisms, but it does have potential drawbacks. First, genes not expressed during host–pathogen interaction will not be detected; however, this is a limitation of RNA-sequencing in general and not specific to this pipeline. Second, functional characterization of genes that lack homologous sequences in public domains may be difficult. We have used BLAST to assay the relatedness of assembled transcripts to known plant or oomycete genes. While this will theoretically generate fewer ambiguous genes, some level of uncertainty is unavoidable, especially if sequences from close relatives are unavailable.

As sequencing technology improves, some fields may reap the benefits more than others. Genomic research on obligate biotrophic pathogens, though rapidly progressing (Hacquard et al., 2013; Zhang et al., 2014; Rudd et al., 2015), still lags behind other phytopathological research. This pipeline streamlines the process of analyzing metatranscriptomic data from plant–pathogen interactions while delivering reliable and meaningful results. Until such time as a complete reference genome is available for each interacting organism, researchers will need to rely upon a combination of careful experimental planning and meticulous data processing and analysis.

## Materials and Methods

### Plant Growth and Infection Assay

Sweet basil ‘Genovese’ seed (Johnny’s Seeds, Lot 48104) was germinated in soil-less growing media (Premier Tech Horticulture PRO-MIX^®^ BX Mycorrhizae^TM^) in a greenhouse propagation room (75°F, 50–60% humidity). Seedlings were transplanted and propagated in 4*”* pots in a plastic house with daytime temperatures reaching 80°F and low relative humidity averaging 20%.

The pathogen *P. belbahrii* was maintained by inoculating basil plants weekly. Basil plants with three sets of true leaves (4–6 weeks old) were inoculated by spraying the leaves thoroughly with water and brushing fresh sporangia from diseased plants onto the wetted abaxial leaf surfaces of new plants. Uninoculated plants were sprayed with distilled water only. Plants were then subjected to 100% humidity by enclosing individual plants in thin plastic for 48 h or until sporulation was visible on inoculated plants. One inoculated plant and one uninoculated plant were randomly selected for RNA-seq analysis.

### RNA Sequencing and Data Analysis

A complete protocol of using the pipeline is attached as Supplementary Material. Total RNAs were extracted from leaves of healthy and infected basil plants using Trizol reagent (Thermo Fisher Scientific, Waltham, MA, USA) following the manufacturer’s protocol. After removal of genomic DNA by DNase I (New England Biolabs, Ipswich, MA, USA) treatment, RNA samples were quantified using NanoDrop 1000 (Thermo Fisher Scientific, Waltham, MA, USA) and assessed for integrity using Agilent Bioanalyzer 2100 (Agilent, Holbrook, MA, USA). Library construction was conducted using Illumina TruSeq mRNA library preparation kit (Illumina, San Diego, CA, USA), followed by sequencing using Illumina HiSeq2000 platform following manufacturer’s protocol. RNA-seq reads quality was examined using FastQC^2^ to determine the necessity of trimming low-quality reads. BAM (Binary SAM) format of RNA-seq data were converted to FASTQ format using bamTofastq command of Bedtools^3^. Paired-end read trimming was conducted by Trimmomatic 0.32 (Bolger et al., 2014) using a sliding window 4 (nucleotide window size):30 (quality score threshold) and excluding reads below a minimal length of 36. The trimmed paired-end reads were examined by FastQC again to confirm improvement of read quality. Trimmed paired-end RNA-seq reads from inoculated and uninoculated plants were pooled and assembled using Trinity in a single run, using 10 Gigabyte of memory on a 10-core CPU computer. The assembled total transcripts (Trinity.fasta) were used as a reference transcriptome. Transcript abundance was estimated for each sample using run_RSEM_align_n_estimate.pl in RSEM_util of Trinity package (RSEM: RNA-seq by Expectation Maximization) (Li and Dewey, 2011) by using trimmed paired-end reads of each sample.

### BLAST and GO Enrichment Analysis

Local BLAST (Basic Local Alignment Search Tool) search was performed using Blast plus (NCBI: National Center for Biotechnological Information^4^) version 2.2.24. A customized oomycete genome database was composed of multiple species including *P*. *infestans*, *P. parasitica*, *P. sojae*, and *Hyaloperonospora arabidopsidis* genomes downloaded from NCBI. Arabidopsis thaliana genome TAIR10 was downloaded from TAIR (The Arabidopsis Information Resource)^5^. The genome database was created using the formatdb command. Pathogen Gene Ontology (GO) enrichment analysis was conducted using the PANTHER^6^ online gene analysis tool. Plant GO enrichment analysis was performed using AgriGO 1.2 following user’s manuals^7^.

### Data and Source Code Access

The RNA-seq data used in this work can be accessed at NCBI GEO (Gene Expression Omnibus) with accession number GSE79807.

## Author Contributions

The project and pipeline were conceived and designed by LG and L-JM. The experiments were performed by LG and AM. Data analysis was performed by LG, YZ, and L-JM. The manuscript was written and revised by LG, GD, KA, AM, RW, and L-JM. The final manuscript was approved by all authors.

## Conflict of Interest Statement

The authors declare that the research was conducted in the absence of any commercial or financial relationships that could be construed as a potential conflict of interest.

## Footnotes

1 http://www.plantgdb.org

2 http://www.bioinformatics.babraham.ac.uk/projects/fastqc/

3 http://bedtools.readthedocs.org/en/latest/

4 ftp://ftp.ncbi.nlm.nih.gov/blast/executables/blast+/2.2.24/

5 https://www.arabidopsis.org/

6 http://www.pantherdb.org/

7 http://bioinfo.cau.edu.cn/agriGO/index.php

## Supplementary Material

SUPPLEMENTARY MATERIAL **A complete protocol for performing metatranscriptomic data analysis using the pipeline**.
