# Supplementary material for: A De Novo-Assembly Based Data Analysis Pipeline for Plant Obligate Parasite Metatranscriptomic Studies
Source: Front Plant Sci. 2016 Jul 11;7:925. doi: 10.3389/fpls.2016.00925 (PMC4939292; doi:10.3389/fpls.2016.00925)
Supplement: SUPPLEMENTARY MATERIAL — A complete protocol for performing metatranscriptomic data analysis using the pipeline. [file Data_Sheet_1.DOCX]

Supplementary Material

**PROTOCOL:**

**A *de-novo-a*ssembly-based Data Analysis Pipeline for Plant Obligate Parasite Metatranscriptomic Studies**

Li Guo^1^, Kelly S. Allen^2^, Greg Deiulio^1^, Yong Zhang^1^, Angela Madeiras^2^, Robert L. Wick^2^, Li-Jun Ma^1^

1. Department of Biochemistry and Molecular Biology

2. Stockbridge School of Agriculture

University of Massachusetts Amherst, Amherst, Massachusetts 01003 USA

**INTRODUCTION**

This protocol describes all necessary procedures in the data analysis pipeline of the article. The objective of this protocol is to discover genes and their expression levels from host and obligate parasite metatranscriptomic sequencing data. A complete visual workflow of this pipeline can be found in the main paper as Figure 1.

**EQUIPMENT**

***Data***: RNA-seq data (fastq or BAM format) collected from sequencing the transcriptome of uninfected plants (control) and a mixed transcriptome (metatranscriptome) of plants infected with an obligate parasite. Illumina sequencing data is recommended. Both single- or pair-end (recommended) reads are acceptable. Inclusion of biological replicates is recommended to prove statistical significance for differentially expressed genes. In this protocol, we use an example dataset of a basil plant with and without infection of *Peronospora belbarhii* for most of data analysis. This data is in two BAM files (infected.bam and control.bam). We also use another example dataset which contains biological replicates to demonstrate the differential gene expression analysis (Step 11).

***Hardware***: 64-bit computer running Linux; ~1 GB of RAM per ~1 million paired-end reads

***Software****:*

- Bowtie: version 1.1.2 (<http://bowtie-bio.sourceforge.net/index.shtml>) (Longmead *et al.* 2009)
- Samtools: version 1.2.1 (<http://www.htslib.org/>) (Li *et al.* 2009)
- FastQC: version 0.11.5 (http://www.bioinformatics.babraham.ac.uk/projects/download.html)
- Trimmomatic: version 0.32 (http://www.usadellab.org/cms/?page=trimmomatic) (Bolger *et al.* 2014)
- bamToFastq (part of Bedtools): version 2.25.0 (<http://bedtools.readthedocs.org/en/latest/>) (Quinlan and Hall, 2010)
- Trinity (includes RSEM): version trinityrnaseq_r2013-02-25 (<http://trinityrnaseq.sourceforge.net>) (Grabherr et al. 2011)
- Blast +: version 2.2.24 (<ftp://ftp.ncbi.nlm.nih.gov/blast/executables/blast+/2.2.24/>) (Altschul *et al.* 1990)
- Java: version 1.7.0_25 (https://java.com/)
- Python: version 2.7 (https://www.python.org/download/releases/2.7/)
- faSomeRecord (http://hgdownload.cse.ucsc.edu/admin/exe/linux.x86_64/)
- R: version 3.2.4 (https://www.r-project.org/)
- edgeR:(<https://bioconductor.org/packages/release/bioc/html/edgeR.html>) (Robinson *et al.* 2010)
- limma:(<https://bioconductor.org/packages/release/bioc/html/limma.html>) (Ritchie *et al.* 2015)
- ggplot2: (<http://ggplot2.org/>) (Wickham 2009)

**PROCEDURES**

1. **Preparing Workspace:**

This very first step is CRITICAL. Before starting data analysis, install all the software listed above in the computer and make sure they are within your Unix PATH settings. It’s best that all software are installed or placed in a single directory called *e.g.* “software” and then explicitly add the directory path (*e.g.* */usr/local/software*) to $PATH variable on your Linux system. For example:

% **export** PATH=$PATH:/usr/local/software

For simplicity, we can define an environmental variable called $TRINITY_HOME to replace the actual path for Trinity, which will be used multiple times in this protocol.

% **export** TRINITY_HOME=/usr/local/software/trinityrnaseq_r2013-02-25

Additionally, a directory should be designated as working directory where raw data files, intermediate files and output files are stored.

% **mkdir** RNA-seq-analysis

% **ls** RNA-seq-analysis

Both BAM files should be in this directory:

control.bam infected.bam

1. **Data quality control**

Raw RNA-seq data needs to be examined for data quality which is usually indicated by quality scores per base and overall distribution. Here we use a software called FastQC to check the data quality:

% **fastqc** control.bam infected.bam

This will generate data quality report on the two bam files:

control_fastqc infected_fastqc

1. **Data format conversion and filtering**

Based on the quality report, a decision will be made whether the data needs to be trimmed for use in the downstream pipeline. Typically a threshold quality score should be chosen for trimming sequences of low quality. Here we use trimmomatic to filter the low quality reads. The standard input data format for trimmomatic is fastq, therefore we need to convert BAM file to fastq files. For example:

% **bamToFastq** -i control.bam -fq control_a.fq -fq2 control_b.fq

% **bamToFastq** -i infected.bam -fq infected_a.fq -fq2 infected_b.fq

“-i”: input bam file

“-fq” first output fastq file

“-fq2” second output fastq file

Then we use *Trimmomatic* to filter the reads (Note: “\” should be removed in actual codes hereafter):

% **java** -jar trimmomatic-0.32.jar PE -phred33 control_a.fq \ control_b.fq control_aPaired.fq control_aUnpaired.fq \ control_bPaired.fq control_bUnpaired.fq LEADING:3 TRAILING:3 \ SLIDINGWINDOW:4:25 MINLEN:36

% **java** -jar trimmomatic-0.32.jar PE -phred33 infected_a.fq \ infected_b.fq infected_aPaired.fq infected_aUnpaired.fq \ infected_bPaired.fq infected_bUnpaired.fq LEADING:3 TRAILING:3 \ SLIDINGWINDOW:4:25 MINLEN:36

This code will trim RNAseq reads using the following criteria: remove leading (TRAILING:3) and trailing (TRAILING:3) low quality or N bases (if quality score below 3); Scan the read with a 4-base wide sliding window (SLIDING WINDOW:4:25), cutting when average quality per base drops below 25; Drop reads below 36 bases long (MINLEN:36).

1. ***de novo* assembly using Trinity** (derived from Hass *et al.* 2013.)

4.1. Read concatenation

Trimmed reads are assembled into transcripts using Trinity. For pathogen transcript discovery, we recommend both control and infected sample pair-end reads are pooled (concatenated) and use Trinity to create a single reference transcript. For example:

% **cat** control_1aPaired.fq infected_1aPaired.fq > left.fq

% **cat** control_1bPaired.fq infected_1bPaired.fq > right.fq

If there are biological replicates (*e.g.* R1, R2, R3), all biological replicates of control and infected conditions can be concatenated:

% **cat** control_R1_left.fq control_R2_left.fq control_R3_left.fq \ infected**_**R1_left.fq infected_R2_left.fq infected_R3_left.fq > left.fq

% **cat** control_R1_right.fq control_R2_right.fq control_R3_right.fq \ infected**_**R1_right.fq infected_R2_right.fq infected_R3_right.fq > \ right.fq

4.2. Trinity assembly

The concatenated data are then *de novo* assembled (Note: Higher settings of RAM and CPU are needed if the data size is big.):

% $TRINITY_HOME/Trinity.pl --JM 40G --CPU 20 --seqType fq --left \ left.fq --right right.fq --output Reference_Trinity

The Trinity.fasta in Reference_Trinity directory is the reference transcripts (pooled reference). We will use it to perform RSEM and estimate FPKM.

4.3. IMPORTANT:

For host plant gene expression analysis (Step 10), another reference (shared reference) should be created by subsetting pooled reference using shared transcripts (a list of transcript IDs) (Figure 2 in main paper):

% faSomeRecord Reference_Trinity/Trinity.fasta shared_transcript.txt \ shared_transcript.fasta

1. **Quantification of transcripts using RSEM**

To quantify expression level of transcripts in control and infected samples, trimmed reads of control and infected samples are mapped to “pooled reference” transcripts:

% $TRINITY_HOME/util/RSEM_util/run_RSEM_align_n_estimate.pl -- \ transcripts Reference_Trinity/Trinity.fasta --seqType fq --left \ control_aP.fq --right control_bP.fq --prefix control \

--thread_count 10 -- --bowtie-chunkmbs 512

% $TRINITY_HOME/util/RSEM_util/run_RSEM_align_n_estimate.pl -- \ transcripts Reference_Trinity/Trinity.fasta --seqType fq --left \ infected_aP.fq --right infected_bP.fq --prefix infected \

--thread_count 10 -- --bowtie-chunkmbs 512

1. **Identify expressed transcripts**

Expressed transcripts (FPKM > 0) can be retrieved using a Unix pipeline such as:

% **cat** control.genes.result |**awk** ‘$7 != 0’|**cut** -f1,7 > \ control_FPKM.txt

% **cat** infected.genes.result |**awk** ‘$7 != 0’|**cut** -f1,7 > \ infected_FPKM.txt

The two outpout files “control_FPKM.txt” and “infected_FPKM.txt” contain gene_ID (column 1) and FPKM values (column 2). To retrieve only gene_IDs, the following pipeline will do:

% **cat** control_FPKM.txt |**cut** -f1 > control_genes.txt

% **cat** infected_FPKM.txt |**cut** -f1 > infected_genes.txt

We will use the two new files to compare transcripts in the next step.

1. **Transcript partition**

To compare two lists of gene_IDs, control_genes.txt, infected_genes.txt, the following script can be used:

First of all, the two lists need to be sorted and duplicates can be removed:

% **sort** control_genes.txt | **uniq** > CK_sorted.txt

% **sort** infected_genes.txt | **uniq** > IN_sorted.txt

% **comm** -23 CK_sorted.txt IN_sorted.txt > control_unique.txt

% **comm** -13 CK_sorted.txt IN_sorted.txt > infected_unique.txt

% **comm** -12 CK_sorted.txt IN_sorted.txt > CK_IN_shared.txt

1. **Transcript subset**

Multiple transcript sequences (subset.fasta) can be retrieved or subset from total transcripts (Trinity.fasta) using “faSomeRecord” function. “genelist.txt” contains a list of gene-ID.

% **faSomeRecord** Trinity.fasta genelist.txt subset.fasta

1. **Functional annotations of transcripts using Local BLAST**

Download genome sequence of organisms to BLAST against. For example, protein sequence of *Arabidopsis thaliana* from TAIR ftp site.

Before doing BLAST, a database has to be created using command formatdb:

formatdb -i Arabidopsis_protiens.fasta -p T

Then transcripts can be BLAST against the database:

blastall -p blastx -i subset.fasta -d Arabidopsis_proteins.fasta -o \ blast_result.txt -e 1e-20 -m 8 -a 10

1. **Differentially expressed gene discovery with biological replicates in bioconductor**

10.1. Run RSEM for control sample biological replicates using shared reference created in Step 4.3. :

% $TRINITY_HOME/util/RSEM_util/run_RSEM_align_n_estimate.pl \

-- transcripts shared_transcript.fasta --seqType fq --left \

control_1aP.fq --right control_1bP.fq --prefix control_R1 \

--thread_count 10 -- --bowtie-chunkmbs 512

% $TRINITY_HOME/util/RSEM_util/run_RSEM_align_n_estimate.pl \

--transcripts shared_transcript.fasta --seqType fq --left control_2aP.fq --right control_2bP.fq --prefix control_R2 \ --thread_count 10 -- --bowtie-chunkmbs 512

% $TRINITY_HOME/util/RSEM_util/run_RSEM_align_n_estimate.pl \

--transcripts shared_transcript.fasta --seqType fq –left \ control_3aP.fq --right control_3bP.fq --prefix control_R3 \

--thread_count 10 -- --bowtie-chunkmbs 512

10.2. Run RSEM for infected sample biological replicates:

% $TRINITY_HOME/util/RSEM_util/run_RSEM_align_n_estimate.pl \

--transcripts shared_transcript.fasta --seqType fq --left \ infected_1aP.fq --right infected_1bP.fq --prefix infected_R1 \

--thread_count 10 -- --bowtie-chunkmbs 512

% $TRINITY_HOME/util/RSEM_util/run_RSEM_align_n_estimate.pl \

--transcripts shared_transcript.fasta --seqType fq --left \ infected_2aP.fq --right infected_2bP.fq --prefix infected_R2 \

--thread_count 10 -- --bowtie-chunkmbs 512

% $TRINITY_HOME/util/RSEM_util/run_RSEM_align_n_estimate.pl \

--transcripts shared_transcript.fasta --seqType fq --left \ infected_3aP.fq --right infected_3bP.fq --prefix infected_R3 \

--thread_count 10 -- --bowtie-chunkmbs 512

10.3. To perform differential gene expression analysis using *edgeR* in bioconductor, we need read count data for transcripts (in isoforms). The following code will convert RSEM results into a read count table:

% $TRINITY_HOME/util/RSEM_util/merge_RSEM_frag_counts_single_table.pl\

control_R1.isoforms.result control_R2.isoforms.result \ control_R3.isoforms.result infected_R1.isoforms.result \ infected_R2.isoforms.result infected_R3.isoforms.result > \ mydata.counts.matrix

10.4. Calculate differentially expressed genes using *edgeR* (Robinson et al., 2010):

First of all, install edgeR package by typing the two lines (one each time) in R:

> source(“https://bioconductor.org/biocLite.R”)

> biocLite(“edgeR”)

Loading *edgeR* package:

> library(edgeR)

Read count_data (first column has a header called “isoforms”):

> mydata <- read.delim("~/mydata.counts.matrix",row.names = "isoforms")

Provide sample information. After the “isoforms” column, the first three columns are control (1) and the following three columns are treatment samples (2):

> group <- factor(c(1,1,1,2,2,2))

Calculate differentially expressed genes:

> Diff <- DGEList(counts=mydata,group=group)

> Diff <- calcNormFactors(Diff)

> design <- model.matrix(~group)

> Diff <- estimateDisp(y, design)

> fit <- glmQLFit(Diff, design)

> qlf <- glmQLFTest(fit, coef=2)

Showing the first few differentially expressed genes:

> topTags(glf)

The output for topTags(glf) looks like this:

logFC logCPM F PValue FDR

comp746720_c0_seq1 13.62494 13.242372 9339.250 2.288523e-202 6.418050e-197

comp747425_c0_seq1 12.12149 13.066616 7759.060 1.737901e-180 2.436928e-175

comp396097_c0_seq1 16.01237 10.078747 2405.222 2.149479e-179 2.009369e-174

comp749225_c0_seq1 15.27920 10.153407 2467.565 6.358895e-179 4.458301e-174

comp748440_c1_seq6 13.96000 10.126923 2368.264 1.621603e-173 9.095409e-169

comp749286_c1_seq1 13.79549 9.848065 2085.537 8.877039e-170 4.149202e-165

comp735583_c0_seq2 12.54245 9.981079 2131.092 4.414905e-165 1.768768e-160

comp748440_c1_seq1 13.08786 9.666081 1885.495 2.274775e-164 7.974367e-160

comp749137_c0_seq1 15.00401 9.068211 1482.866 3.499832e-161 1.090567e-156

comp749320_c0_seq1 14.27108 9.133886 1514.754 9.320242e-161 2.613815e-156

Store the differentially expressed genes in a vector called “DEGs” which contains first 50000 rows, sorted by p.values and using a cutoff of 0.05:

> DEGs <- topTags(qlf,n = 50000,adjust.method = "BH",p.value = \ 0.05,sort.by = "PValue")

Finally, write the DEGs into a tab-delimited file called “DEGs.txt” and export it to local PC:

> write.table(DEGs, file="DEGs.txt",sep=”\t”, quote = FALSE, row.names

= TRUE)

**References**:

Altschul, S.F., Gish, W., Miller, W., Myers, E.W. & Lipman, D.J. (1990) "Basic local alignment search tool." J. Mol. Biol. 215:403-410.

Bolger, A.M., Lohse, M., and Usadel, B. (2014). Trimmomatic: a flexible trimmer for Illumina sequence data. *Bioinformatics* 30, 2114-2120.

Grabherr, M.G., Haas, B.J., Yassour, M., Levin, J.Z., Thompson, D.A., Amit, I., et al. (2011). Full-length transcriptome assembly from RNA-seq data without a reference genome. *Nat Biotech* 29**,** 644-652. doi:10.1038/nbt.1883.

Haas, B.J., Papanicolaou, A., Yassour, M., Grabherr, M., Blood, P.D., Bowden, J., Couger, M.B., Eccles, D., Li, B., Lieber, M., Macmanes, M.D., Ott, M., Orvis, J., Pochet, N., Strozzi, F., Weeks, N., Westerman, R., William, T., Dewey, C.N., Henschel, R., Leduc, R.D., Friedman, N., and Regev, A. (2013). *De novo* transcript sequence reconstruction from RNA-seq using the Trinity platform for reference generation and analysis. *Nat. Protocols* 8**,** 1494-1512.

Wickham H. ggplot2: Elegant Graphics for Data Analysis. (2009) Springer-Verlag New York.

Langmead, B., Trapnell, C., Pop, M., and Salzberg, S.L. (2009). Ultrafast and memory-efficient alignment of short DNA sequences to the human genome. *Genome Biology* 10**,** 1-10.

Li H., Handsaker B., Wysoker A., Fennell T., Ruan J., Homer N., Marth G., Abecasis G., Durbin R. and 1000 Genome Project Data Processing Subgroup (2009) The Sequence alignment/map (SAM) format and SAMtools. Bioinformatics, 25, 2078-9.

Quinlan, A.R., and Hall, I.M. (2010). BEDTools: a flexible suite of utilities for comparing genomic features. *Bioinformatics* 26**,** 841-842.

Ritchie ME, Phipson B, Wu D, Hu Y, Law CW, Shi W and Smyth GK (2015). “limma powers differential expression analyses for RNA-sequencing and microarray studies.” *Nucleic Acids Research*, **43**(7), pp. e47.

Robinson, M.D., Mccarthy, D.J., and Smyth, G.K. (2010). edgeR: a Bioconductor package for differential expression analysis of digital gene expression data. *Bioinformatics* 26**,** 139-140.

**
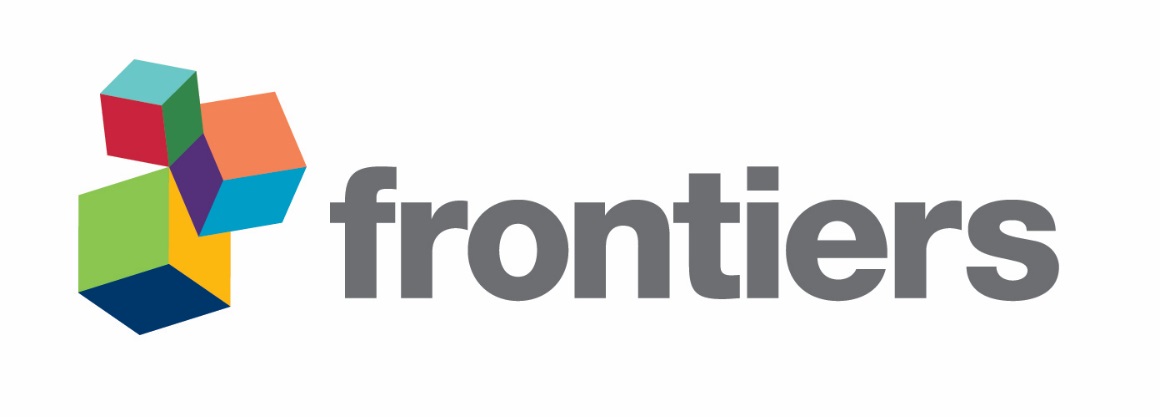
**
